# Improving patient understanding of prenatal screening tests: Using naturally sampled frequencies, pictures, and accounting for individual differences

**DOI:** 10.1016/j.pecinn.2023.100197

**Published:** 2023-08-08

**Authors:** Lauren M. West, Gary L. Brase

**Affiliations:** Department of Psychological Sciences, Kansas State University, Manhattan, KS, USA

**Keywords:** Testing communication, Decision making, Genetic counseling, Health literacy, Prenatal diagnosis, Risk assessment

## Abstract

**Objective:**

Health professionals must communicate prenatal screening test results effectively to patients, but these tests involve very low prevalence and high false positive risks; a situation known to be difficult for people to understand.

**Methods:**

The present experiments evaluated the effectiveness of presenting prenatal screening test results for Trisomy 21, Trisomy 13, or DiGeorge Syndrome, using either naturally sampled frequencies or standard percentages. Participants were given a task of interpreting the posterior probability of an embryo having the condition.

**Results:**

People were significantly better with naturally sampled frequencies. Numerical literacy and visuospatial ability significantly accounted for individual differences in performance within conditions. Participants nevertheless did not differ in ratings of how useful the different presentation formats were, suggesting a lack of awareness of how format influenced understanding. These results held regardless of whether the respondents were undergraduates (Experiment 1) or members of the general population recruited online (Experiment 2).

**Conclusion:**

Using naturally sampled frequencies improves patient understanding of prenatal screening tests results, with low cost of implementation.

**Innovation:**

Using realistic prenatal screening test results, these results show how to improve patient counseling via the use of naturally sampled frequencies.

## Highlights

• Prenatal screening test results are both high stakes and challenging for patients.
• Prior work indicates naturally sampled frequencies can improve this understanding.
• Natural frequencies improved understanding of realistic prenatal screening results.
• Participants did not, however, have insight into this differential usefulness.

## 1 Introduction

The stakes involved in genetic counseling are arguably among the highest in the medical counseling field; the majority of women referred for prenatal screening due to risk factors indicate they would choose to terminate a pregnancy that tested positive for a disability [21,22]. There are therefore both substantial benefits that come with knowing one's genetic information as well as potential negative social and psychological implications that come with that information and subsequent decisions [26]. As the field of genetic testing grows, the issues of more effective and individualized treatment become more important. The understanding of medical test information is an essential first step to early intervention and individualized treatment of a genetic disease [20]. With this in mind, there are significant benefits to patients' accurate understanding of genetic counseling information.

Counselors need to do all they can to ensure that the information they provide is accurately understood by patients, which includes the base-rates of different genetic conditions, the sensitivity (true-positive) rate of the test, the specificity (1-false-positive) rate of the test, and how these statistics all relate with one another. Beyond these quantitative topics counselors also ideally should help patients work through their evaluations of the potential benefits and harms of taking – or not taking— the test at all.

It is therefore imperative that patients understand what they are being told, and that genetic counselors work with patients to ensure their understanding of the probability of their child having a genetic disease. These are situations calling for Bayesian inference (determining the posterior probability of an event, given some new information to be combined with an initial base rate), which are common in health and medicine.

There are indications that genetic counselors are not always effectively communicating risk to their patients [2]. At the same time, however, there are very limited studies focused on risk communication *specifically within the genetic counseling field*. The present research was designed to improve client/counselor communication about prenatal genetic screening tests results by providing clear and concrete results on people's understanding of those results when given different result presentation formats. Our hope is that the most effective of these formats will provide a foundation for more effective and efficient genetic counseling practices.

This issue is significant because adopting these types of presentation formats can immediately improve the effective health literacy of patients across a wide array of medical fields. For example, the use of particular numerical formats to improve the communication of medical information has been advocated before, but often in general terms and not targeted to specific medical specialties in terms of content or audience (e.g., [12,17]). These general recommendations include that complex medical test results should use frequencies (i.e., whole numbers), present those frequencies within a natural sampling framework (e.g., [13,17]; for a review and meta-analysis, see [30]), and supplement the numerical text results with a graphical presentation of the results such as a pictograph [4,5,9,23].

The above presentation formats are used in a genetic counseling context in Germany,^1^ following a first-trimester screening procedure. Adoption beyond this one country could be facilitated by research on the efficacy of these presentation formats, in this particular domain. Genetic counseling involves specific types of screening tests, typically focused on genetic conditions with very low base rates in the overall population and therefore subject to relatively high false positive impacts, even with very good specificity. For example, non-invasive prenatal tests (NIPT) for Down Syndrome generally find 417 positives in a pool of 100,000 pregnancies. Of these 417 positive test results, 97 of them are expected to be false positives [28]. It is this kind of information that can affect the patient's understanding of their test results.

Additionally, recent research has also begun to focus on the role of individual differences in understanding complex statistical reasoning situations, such as medical test results. Numerical literacy is a major part of overall health literacy [1,14,15], and similar roles have recently been studied for visuospatial abilities, graph literacy, and cognitive reflection abilities [6,7]. Although the prior literature on numerical literacy and other individual differences as predictors of statistical reasoning performance is somewhat conflicted, most studies find that numeracy and some of the above other factors significantly account for individual differences in Bayesian reasoning. In the present context, of course, attending to the individual differences between different clients is an important aspect of tailoring genetic counseling to the needs and abilities of particular clients.

## 2 Experiment 1

The present research evaluates people's abilities to correctly understand and interpret medical test results, and in particular reach the posterior probability of a genetic disorder (incorporating base rates and test results). Importantly, the materials for these studies are taken from genetic counseling. The medical test results used for this research are screening tests for Trisomy 21, DiGeorge Syndrome, and Trisomy 13. These conditions vary in prevalence, although they are all fairly uncommon, and the screening tests generally have both false positive and false negative rates associated with them. Thus, these are very difficult, yet realistic, test results that genetic counselors are asked to help walk clients through. Furthermore, it can be tempting to use percentages for prevalence because it conveys the rarity of conditions, even as it can be misleading for communicating relative false positive results likelihoods.

We hypothesize that specific factors will improve understanding, and thereby quantitative reasoning, about the prenatal genetic screening tests. Specifically:

1) Naturally sampled frequencies will improve statistical reasoning performance;
2) Numerical literacy, and possibly other individual differences (visuospatial abilities, graph literacy, and cognitive reflection), will predict statistical reasoning performance, independent of presentation format.

These studies also include follow-up items about understanding and subjective responses to the statistical reasoning tasks. Although these are more exploratory, based on Brase [3], we expect that the use of naturally sampled frequencies will have a positive effect on these evaluations.

### 2.1 Methods

#### 2.1.1 Participants

Data were collected from 431 undergraduates at a large public university. For their participation, students received credit toward partial fulfillment of their introductory psychology course or extra credit in an upper-level psychology course. After eliminating 32 people who did not complete the entire study, the remaining 399 participants were used for analyses.

This sample size was based on prior research [7] and a goal of sufficient power for similarly sized effects (i.e., for a small/medium effect size *ɳ* of 0.35 and a power of 0.80) using a z-test of proportions. The average age of participants was 19.2 years (SD of 2.59), and 136 of the participants (34.1%) were male.

#### 2.1.2 Materials and procedures

**Fig. 1:**
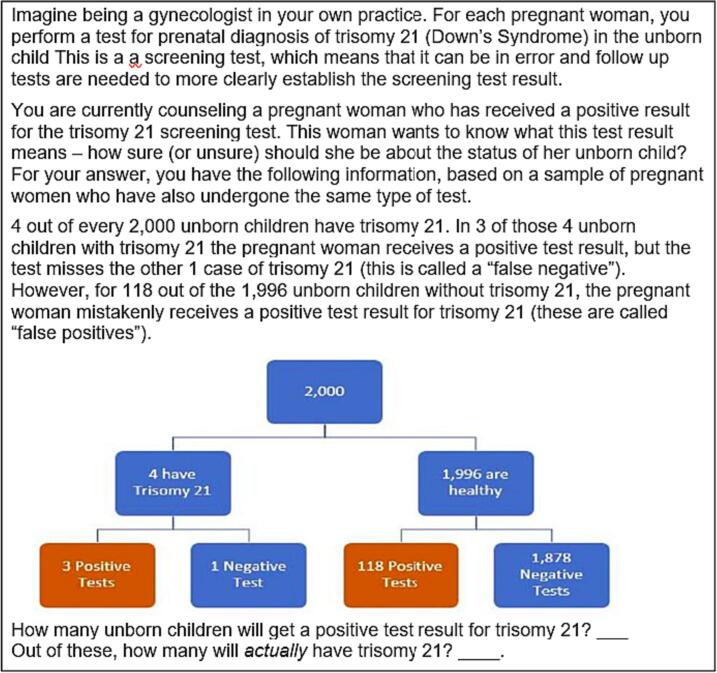
Example of the Bayesian reasoning task in Experiment 1; Trisomy 21 with naturally sampled frequencies.

The protocol for this and all subsequent experiments was reviewed and declared exempt by the relevant Institutional Review Board and were implemented using an online survey platform (Qualtrics). The basic methodology of the studies followed prior research (e.g., [7]), in that participants were randomly given a statistical reasoning tasks from a set that were isomorphic other than key variable manipulations in a between-subjects design. Unlike in prior Bayesian reasoning research, which used arbitrary medical scenarios (or didn't use medical scenarios at all), this research used genetic counseling scenarios and typical test performance information. Specifically, the Bayesian reasoning task used here involved, in a between-subjects design, one of three different realistic genetic counseling contexts (Trisomy 21, Trisomy 13, and DiGeorge Syndrome), with participants receiving either standard (normalized) percentage information or naturally sampled frequency information. Participants in all of the conditions also received a pictorial aid as part of the task that showed the key numbers in a nested tree form. Fig. 1 shows an example condition. See Supplemental Materials and https://osf.io/pujr6/ for larger images and full text of all these tasks.

The contexts for the Bayesian reasoning tasks (Trisomy 21, Trisomy 13, and DiGeorge Syndrome) all have very low base rates and relatively high false positive rates, but they vary in rarity (how low the base rates are; see Table 1). Because this particular Bayesian reasoning context is known, based on prior research, to be particularly difficult for people the responses were kept in the format of naturally sampled frequencies (i.e., asking for the total number of positive results and true positives). Numbers for the tasks were drawn from published and online government sources [28]. After the reasoning task, participants were given two follow-up questions about the reasoning task. One asked, “You just answered a question about how to understand a prenatal test for a genetic condition. How useful do you think this test is for expectant parents?” (rated on a 0–100 slider scale). The second follow-up item asked participants to “Please explain how you reached the answers you gave for the previous problem about genetic testing,” followed by an open response text box.

**Table 1:** Base rates, true positive rates, and false negative rates for each genetic screening test used in the Bayesian reasoning tasks.

		Information (naturally sampled frequencies)						Bayes
*H*	*D*	*H*		*D|H*		*D| ~ H*		*p(H|D)*
Trisomy 21	Screening test	4	2000	3	4	118	1996	3/121 (0.025)
DiGeorge Syndrome	Screening test	4	8000	3	4	40	7996	3/43 (0.070)
Trisomy 13	Screening test	12	60,000	11	12	58	59,988	11/69 (0.159)

A third section consisted of four measures of individual differences and ability, presented in random order and with randomized item orders within each measure. These are all measures that have been found in prior research to be predictive of statistical reasoning task performance (e.g., [6,7,19,24,27,30])

a) The multiple-choice version of the general numeracy scale (MCQ-GNS; [16]), which consists of 11 items, each with the most common four answers as alternatives. For example, one item in this scale is, “Which of the following represents the biggest risk of getting a disease?” (0.8%, 1%, 5%, or 10%), and an overall score for each individual was a tally of correct answer choices. The MCQ-GNS showed weak reliability (Cronbach's α = 0.65), and the present sample had a mean of 8.35 and standard deviation of 2.12.
b) The Paper Folding Test of visuospatial ability [10], which consists of 20 items that each contains a series of drawings of a uniquely folded square piece of paper with a hole punched through the folded paper in a specific location. For each drawing, participants are then asked to judge what the folded paper would look like, once unfolded, by selecting one of five pictures of unfolded papers located to the right of the original drawings. The Paper Folding Test showed good reliability (Cronbach's α = 0.87), and the present sample had a mean of 12.0 and standard deviation of 4.77.
c) The integrated, verbal Cognitive Reflection Task (CRTverbal) uses non-mathematical tasks from two sources [25,29] for a total of 13 items. The CRTverbal is designed to assess ability to understand and deliberatively reason about language-based riddles, and crucially does not also involve numerical literacy. For example, one item in this scale is, “Mary's father has 5 daughters but no sons – Nana, Nene, Nini, Nono. What is the fifth daughter's name probably?” The CRTverbal showed good reliability (Cronbach's α = 0.83), and the present sample had a mean of 7.69 and standard deviation of 3.30.
d) The Graph Literacy test [11], which consists of 13 items that are either multiple choice or ask for a numeric response. All the items reference accompanying graphs and ask basic questions about interpretation (e.g., For example, “Approximately what percentage of people had Adeolitis in the year 2000?” An overall score for each individual was a tally of correct answer choices. As in prior research (Brase, under review), the Graph Literacy test showed poor reliability (Cronbach's α = 0.39). The present sample had a mean of 10.2 and standard deviation of 1.64.

Following these measures, participants were presented with a page that thanked them for participation and provided debriefing information.

### 2.2 Results

Performance on the Bayesian reasoning task was coded as correct if a participant gave the exact correct answer (both the total positive and true positive rates, which together can be used to express the posterior probability value), but leniency was given for the order in which the numbers were entered (i.e., the smaller number was treated as the true positive rate and the larger number as the total positive rate). As shown in Table 2, performance indicating a correct understanding of these prenatal test results was almost entirely absent when the information was given as percentages, but almost 30% of people showed correct Bayesian reasoning when the same test results were given as naturally sampled frequencies. This difference in performance (statistically significant for each context story, with one-sided difference of proportion tests of *z* = 4.22–4.68, *p* < .001, phi effect sizes 0.37–0.41) is all the more remarkable because people's ratings of how useful the test information showed no significant difference (see Table 2b; *t*(134) = 0.06, *p* = .95, Cohen's *d* = 0.01; *t*(130) = 0.71, *p* = .478, Cohen's *d* = 0.12; *t*(129) = 1.88, *p* = .062, Cohen's *d* = 0.33). Analyses were conducted using Jamovi project [18] and the data for all experiments are available at https://osf.io/pujr6/.

**Table 2:** a and b. Bayesian reasoning performance (proportion reaching correct answer) for different types of prenatal test results, presented as either percentages or natural frequencies information (Table 2), and ratings of how useful the information was judged by reasoners (Table 2b; standard deviations shown in parentheses).

	Percentages	Natural Frequencies
Trisomy 21	0.015	0.273
DiGeorge Syndrome	0.00	0.294
Trisomy 13	0.00	0.282


	Percentages	Natural Frequencies
Trisomy 21	58.5 (24.8)	58.7 (26.2)
DiGeorge Syndrome	50.7 (28.3)	59.2 (23.0)
Trisomy 13	60.6 (24.7)	63.5 (22.4)

Given the consistency of the results across the different prenatal contexts, further analyses combined across contexts. Looking at the individual difference measures, all the measures were intercorrelated (*r =* 0.26–0.49; see Table S1 in Supplemental Materials), and all of them were also significantly correlated with Bayesian reasoning performance (*r =* 0.13–0.25; see Table S1 in Supplemental Materials). This led us to focus on identifying the more foundational predictors from the set of variables and to concurrently evaluate possible multicollinearity issues.

To help clarify which individual differences were primary predictors of Bayesian reasoning performances, two binomial logistic regressions were conducted, one for tasks using percentages information and the other for tasks using natural frequencies, both using all the individual difference measures as potential predictor variables and Bayesian reasoning task performance as the target variable (full regression results are provided in the Supplemental Materials, Tables S2 and S3).

Bayesian reasoning performance when the task included percentages was not significantly predicted by any variables (*X*^*2*^(4) = 1.27, *p* = .866, adjusted R^2^_McF_ = of 0.10). This is likely due to the exceptionally few people who reached the correct response. In contrast, performance when the task included natural frequencies was significantly predicted by numeracy and visuospatial ability; (*X*^*2*^(4) = 39.9, *p* < .001, adjusted R^2^_McF_ = of 0.12. Looking at the variance inflation factors (VIF) as an indication of multicollinearity, these were all below 1.5 and therefore the significant predictors are not problematically multicollinear.

## 3 Experiment 2

Experiment 1 found that Bayesian reasoning about prenatal screening test results were consistently better when information was presented as naturally sampled frequencies rather than percentages, controlling for the context, quantities involved, and type of pictorial aids. People did not have a strong insight into how much information format changed their performance, with similar ratings of the information usefulness of both formats, although they did rate the natural frequencies as slightly more useful. Lastly, numerical literacy and visuospatial ability were significant predictors of statistical reasoning performance (consistent with prior work; [7]), whereas graph literacy and cognitive reflection were not.

For these results to be confidently applied to the field of genetic counseling, it is important to demonstrate that they are reliable. Even further, it would be good to demonstrate that these findings are robust: they occur even with a more diverse, non-college student population. Experiment 2 therefore sought to replicate Experiment 1 with a broader and more population-representative sample. The hypotheses and methodology of this research parallel those of Experiment 1.

### 3.1 Methods

As in Experiment 1, participants were asked to evaluate and answer statistical reasoning tasks in the context of realistic genetic counseling tests, with some participants receiving tasks that use either percentages (standardized relative frequencies) or naturally sampled frequencies.

#### 3.1.1 Participants

Data were collected from 818 people recruited online through a research participation platform (CloudResearch). For their participation, they received $0.75 and they had up to one hour to take the study, which was expected to take about 30 min to complete. The average time to complete the study was 22.2 min. To ensure that participants were attentive and fully engaged in the study, several screening tools were applied for inclusion of the data for analysis. These included a Captcha, an English competency question (reading a short passage and answering a question pertaining to its meaning), and at the end of the survey, another pair of questions were used to check that participants were still paying attention. Only data from participants who passed all these checks were used, resulting in 429 validated participants (52%) who were used for analyses. The average age of participants was 44.0 years (SD of 13. 9), and 122 of the participants (28.4%) were male.

#### 3.1.2 Materials and procedures

The materials and procedure of this study was the same as Experiment 1, save that the third section was reduced to the two measures of individual differences and ability which were significant predictors in the prior study:

a) The multiple-choice version of the general numeracy scale (MCQ-GNS; [16]), which showed weak reliability (Cronbach's α = 0.64, and the present sample had a mean of 9.03 and standard deviation of 1.84.
b) The Paper Folding Test of visuospatial ability [10], which showed good reliability (Cronbach's α = 0.85), and the present sample had a mean of 9.96 and standard deviation of 4.68.

Following these measures, participants were presented with a page that thanked them for participation and provided debriefing information.

### 3.2 Results

Performance on the Bayesian reasoning task was coded in the same way as in Experiment 1, and Table 3a shows that performance was similar to the previous study. In fact, Bayesian reasoning was higher than in Experiment 1 when the same test results were given as naturally sampled frequencies (possibly due to the more stringent data quality screening). Performance when information was given as percentages remained near zero, which was again significantly lower (*z* = 5.34–5.88, *p* < .001, phi effect sizes 0.45–0.48). As in Experiment 1, ratings of how useful the natural frequencies versus percentages test information was showed no recognition of the beneficial impact of natural frequencies (*t*(427) = 0.52, *p* = .515, Cohen's *d* = 0.06) Analyses were conducted using Jamovi project [18] and the data for all experiments are available at https://osf.io/pujr6/.

**Table 3:** a and b. Bayesian reasoning performance (proportion reaching correct answer) for different types of prenatal test results, presented as either percentages or natural frequencies information (Table 2), and ratings of how useful the information was judged by reasoners (Table 2b; standard deviations shown in parentheses).

	Percentages	Natural Frequencies
Trisomy 21	0.00	0.365
DiGeorge Syndrome	0.015	0.387
Trisomy 13	0.00	0.402


	Percentages	Natural Frequencies
Trisomy 21	64.3 (28.1)	63.3 (26.5)
DiGeorge Syndrome	61.4 (24.5)	58.1 (29.5)
Trisomy 13	68.9 (27.4)	67.8 (24.0)

As in Experiment 1, the consistency of the results across the different prenatal contexts led to further analyses being combined across these. Numeracy correlated with visuospatial ability (*r* = 0.43, *p* < .001) and both were correlated with Bayesian reasoning performance (*r* = 0.23, *p* < .001 and *r* = 0.28, *p* < .001, respectively). This led us to focus on identifying the more foundational predictors from the set of variables and to concurrently evaluate possible multicollinearity issues.

Binomial logistic regressions were conducted to identify primary predictors of Bayesian reasoning performances with either percentages or natural frequency information (full regression results are provided in the Supplemental Materials, Tables S4 and S5). Bayesian reasoning performance when the task included percentages was not significantly predicted by any variables (*X*^*2*^(2) = 3.11, *p* = .211, adjusted R^2^_McF_ = 0.21). This is likely due to the exceptionally few people who reached the correct response. In contrast, performance when the task included natural frequencies was significantly predicted by numeracy and visuospatial ability; (*X*^*2*^(2) = 43.5, *p* < .001, adjusted R^2^_McF_ = 0.10. Looking at the variance inflation factors (VIF) as an indication of multicollinearity, these were all below 1.2 and therefore the significant predictors are not problematically multicollinear.

## 4 Discussion and conclusion

### 4.1 Discussion

Although the use of naturally sampled frequencies significantly improved the understanding of prenatal genetic screening test results, not everyone benefitted equally from this use. In particular, numerical literacy and visuospatial ability were significant predictors of reasoning outcomes. To illustrate this, consider that people with numeracy one standard deviation below the mean in Experiment 2 (*n* = 38) gave the correct Bayesian reasoning answer –even when given natural frequencies information – only 10.5% of the time. On the other hand, people with numeracy one standard deviation above the means (and with natural frequency information, *n* = 56) gave the correct answer 62.5% of the time.

Ensuring that patients in genetic counseling understand their medical results is thus a function of both how that information is presented and patients' abilities to use that information (i.e., numerical literacy). The present research provides some guidance for both of these factors; how to present prenatal screening test results and some important factors to consider when anticipating how much counseling will be needed to communicate those results [2,20,21,26].

Contrary to our expectations, the facilitative effect of natural frequencies was not reflected in participants' evaluations of how useful they thought the test was. Similarly, the open text responses of participants about how they reached the answer to their screening test problem did not appear to indicate any systematic insights or procedural distinctions across the different experimental conditions (text responses are available in the open data at https://osf.io/pujr6/). There may be some further clarification to do regarding these exploratory measures (e.g., ensuring that people interpret “useful” as referencing the test information presentation, distinct from the test diagnosticity) but at this point it appears that there is little guidance from patient intuition about the correct interpretation of screening test results.

Additional research is needed to address limitations of these studies. Actual genetic counseling is, of course, different from the setting of these two experiments. One could presume that actual patients are more highly motivated to fully understand their test results, and this will generally improve performance [9]. Additionally, genetic counseling often includes a process of reviewing and explaining test results with patients, which is quite different from the simple presentation of text used here. That setting would likely also help patients in reaching a good understanding of the test results. One way of addressing these weaknesses could be to use screening test presentations to actual patients but, given the now well-documented issues with the use of percentage results information, including those conditions could be ethically problematic in actual counseling.

### 4.2 Innovation

The two experiments reported here provide clear and direct guidance for how to better present genetic screening test results, particularly in the context of counseling prospective parents. Giving information in naturally sampled frequencies and using a pictorial summary greatly improves understanding of the results. The present research is innovative in that it demonstrates these effects using content that is representative of genetic screening test results.

To be clear, these presentation guidelines are not sufficient to induce everyone (or even the overall majority) to reach the correct answer, indicating a full understanding of these screening tests. Individual differences in ability and motivation (e.g., numerical literacy and visuospatial ability) play significant roles as well. Without naturally sampled frequencies, though, almost no one reached the correct answer. These presentation guidelines are therefore necessary foundations to start from and using percentages as a format for presenting results cannot be recommended.

### 4.3 Conclusion

The present research demonstrates that using naturally sampled frequencies and visual representations of test results improves patient understanding of prenatal screening tests results, with low cost of implementation. These results are consistent with a large body of evidence from more general contexts on these formats as aids to Bayesian reasoning (e.g., [7,13,14]). Along with examples from the German health care system, the barriers to adoption of these presentation methods are very low. This format does not guarantee patient understanding, as other factors (e.g., numerical literacy, visuospatial ability) also play roles in its effectiveness, but it makes understanding easier and more likely.

## Declaration of Competing Interest

The authors declare that they have no known competing financial interests or personal relationships that could have appeared to influence the work reported in this paper.

## Appendix A Supplementary data

Study materials and analysis details. Image 1

## Footnotes

1 See: https://www.g-ba.de/downloads/40-268-7477/2021-04-22_Mu-RL_Einleitung-SN-Versicherteninfo-NIPT_Anlage-3-Versicherteninof-spezifisch.pdf and https://www.gwq-serviceplus.de/faktenboxen/NIPT/gwq_faktenbox_nipt.pdf
