# Supplementary material for: Improving patient understanding of prenatal screening tests: Using naturally sampled frequencies, pictures, and accounting for individual differences
Source: PEC Innov. 2023 Aug 8;3:100197. doi: 10.1016/j.pecinn.2023.100197 (PMC10429283; doi:10.1016/j.pecinn.2023.100197)
Supplement: Supplementary file 1 — Study materials and analysis details. [file mmc1.docx]

**Bayesian Reasoning Tasks**

**Instructions**

The following items ask for your judgments about what the outcome will be in a certain situation. Each question includes several pieces of information and ends with a question for you to answer.  These are supposed to be very difficult problems, so do not feel badly if they are hard for you to answer.  Please just do your best, and provide as good an answer as you can.  
*Please give your answer in the space provided. If you believe that the answer may change each time the situation described occurs, please give us what you believe the “typical” outcome will be. You may use paper and pencil to write out calculations, as long as you put your final answer in the space provided.*

| Imagine being a gynecologist in your own practice. For each pregnant woman, you perform a test for prenatal diagnosis of trisomy 21 (Down’s Syndrome) in the unborn child This is a a screening test, which means that it can be in error and follow up tests are needed to more clearly establish the screening test result.  You are currently counseling a pregnant woman who has received a positive result for the trisomy 21 screening test. This woman wants to know what this test result means – how sure (or unsure) should she be about the status of her unborn child?  For your answer, you have the following information, based on a sample of pregnant women who have also undergone the same type of test. | |
| --- | --- |
| **[Natural Frequencies]**  4 out of every 2,000 unborn children have trisomy 21. In 3 of those 4 unborn children with trisomy 21 the pregnant woman receives a positive test results, but the test misses the other 1 case of trisomy 21 (this is called a “false negative”). However, for 118 out of the 1,996 unborn children without trisomy 21, the pregnant woman mistakenly receives a positive test result for trisomy 21 (these are called “false positives”). | **[Percentages]**  0.2% of unborn children have trisomy 21. In 75% of unborn children with trisomy 21 the pregnant woman receives a positive test results, but the test misses the other 25% of cases of trisomy 21 (this is called a “false negative”). However, for 5.9% of the unborn children without trisomy 21, the pregnant woman mistakenly receives a positive test result for trisomy 21 (these are called “false positives”). |
| The picture below shows the same information in a picture.  The top row starts with the total sample, and the second row shows how many unborn children have the condition.  The third row shows how many in each group test positive (orange boxes) or negative. | |
| 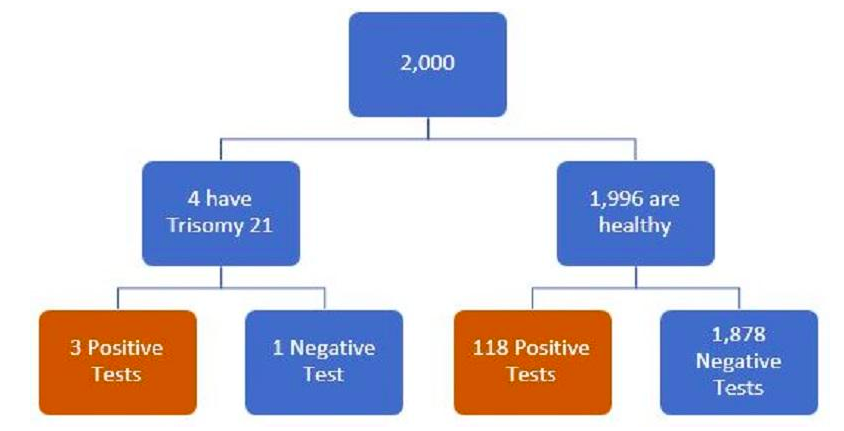 | 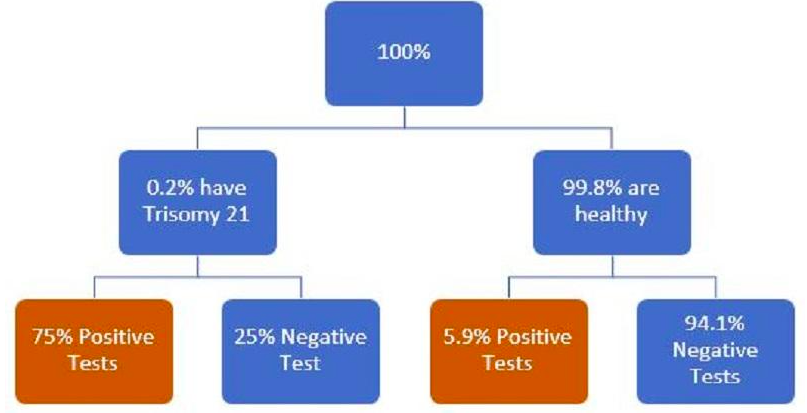 |
| (Please fill in the correct numbers)  How many unborn children will get a positive test result for trisomy 21?  _____  Out of these, how many will *actually* have trisomy 21?    _____ | |

| Imagine being a gynecologist in your own practice. For each pregnant woman, you perform an NIPT (Non-Invasive Prenatal Test) screening test for prenatal diagnosis of genetic issues. The NIPT is a screening test; which means that it can be in error, and follow up tests are needed to more clearly establish the NIPT result.  You are currently counseling a pregnant woman who has received a positive NIPT test result for DiGeorge Syndrome—a deletion on the 22nd chromosome that has very severe effects. This woman wants to know what this test result means – how sure (or unsure) should she be about the status of her unborn child?  For your answer, you have the following information, based on studies of DiGeorge Syndrome and the NIPT test as a screening test for this condition. | |
| --- | --- |
| **[Natural Frequencies]**  About 4 out of every 8,000 unborn children have DiGeorge Syndrome.In 3 of those 4 unborn children with DiGeorge Syndrome, the pregnant woman receives a positive NIPT screening test result, but the NIPT misses the other 1 case of DiGeorge Syndrome (this is called a “false negative”).Also, for 40 out of the 7,996 unborn children without DiGeorge Syndrome, the pregnant woman mistakenly receives a positive NIPT test result for DiGeorge Syndrome (these are called “false positives”). | **[Percentages]**  About 0.05% of unborn children have DiGeorge Syndrome.In 75% of unborn children with DiGeorge Syndrome, the pregnant woman receives a positive NIPT screening test result, but the NIPT misses the other 25% of cases of DiGeorge Syndrome (these are called “false negatives”).Also, for 0.5% of unborn children without DiGeorge Syndrome, the pregnant woman mistakenly receives a positive NIPT test result for DiGeorge Syndrome (these are called “false positives”). |
| The picture below shows the same information in a picture. The top row starts with the total sample, and the second row shows how many unborn children have the condition. The third row shows how many in each group test positive (orange boxes) or negative. | |
| 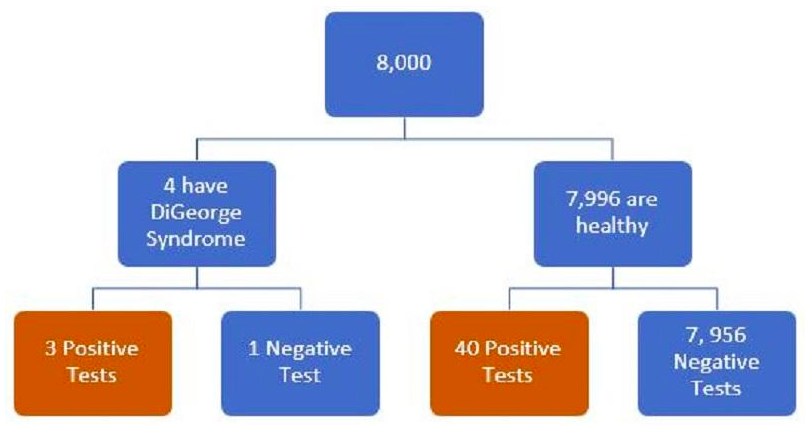 | 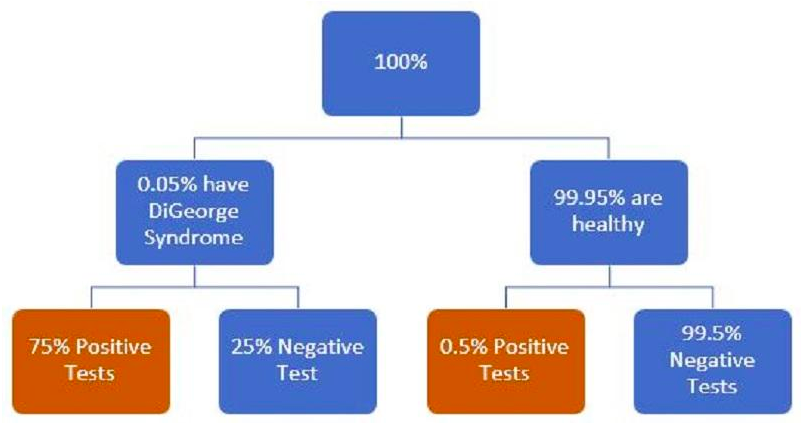 |
| (Please fill in the correct numbers) How many unborn children will get a positive NIPT test for DiGeorge Syndrome?  _____  Out of these, how many will *actually* have DiGeorge Syndrome?  _____ | |

| Imagine being a gynecologist in your own practice. For each pregnant woman, you perform an NIPT (Non-Invasive Prenatal Test) screening test for prenatal diagnoses of genetic issues. The NIPT is a screening test; which means that it can be in error, and follow up tests are needed to more clearly establish the NIPT result.  You are currently counseling a pregnant woman who has received a positive NIPT test result for trisomy 13 (Patau Syndrome), which has very severe effects. This woman wants to know what this test result means – how sure (or unsure) should she be about the status of her unborn child?  For your answer, you have the following information, based on studies of trisomy 13 and the NIPT test as a screening test for this condition. | |
| --- | --- |
| **[Natural Frequencies]**  About 12 out of every 60,000 unborn children have trisomy 13.In 11 of those 12 unborn children with trisomy 13, the pregnant woman receives a positive NIPT screening test result, but the NIPT misses the other 1 case of trisomy 13 (this is called a “false negative”).Also, for 58 out of the 59,988 unborn children without trisomy 13, the pregnant woman mistakenly receives a positive NIPT test result for trisomy 13 (these are called “false positives”). | **[Percentages]**  About 0.02% of unborn children have Trisomy 13.In 92% of unborn children with Trisomy 13, the pregnant woman receives a positive NIPT screening test result, but the NIPT misses the other 8% of cases of Trisomy 13 (these are called “false negatives”).Also, for 0.097% of unborn children without Trisomy 13, the pregnant woman mistakenly receives a positive NIPT test result for Trisomy 13 (these are called “false positives”). |
| The picture below shows the same information in a picture. The top row starts with the total sample, and the second row shows how many unborn children have the condition. The third row shows how many in each group test positive (orange boxes) or negative. | |
| 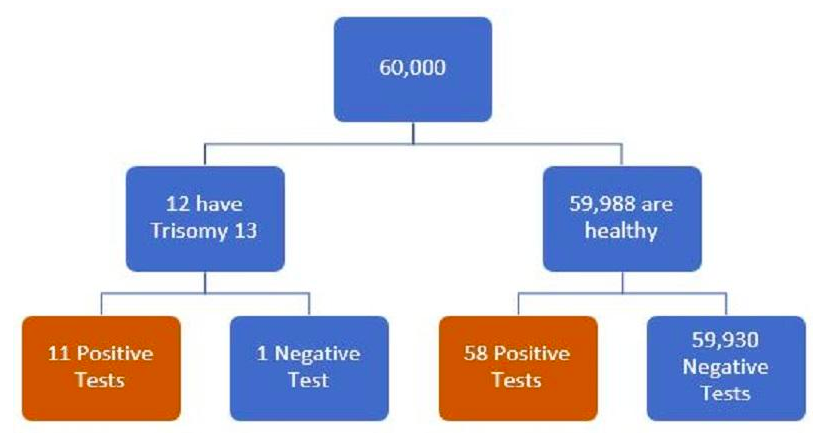 | 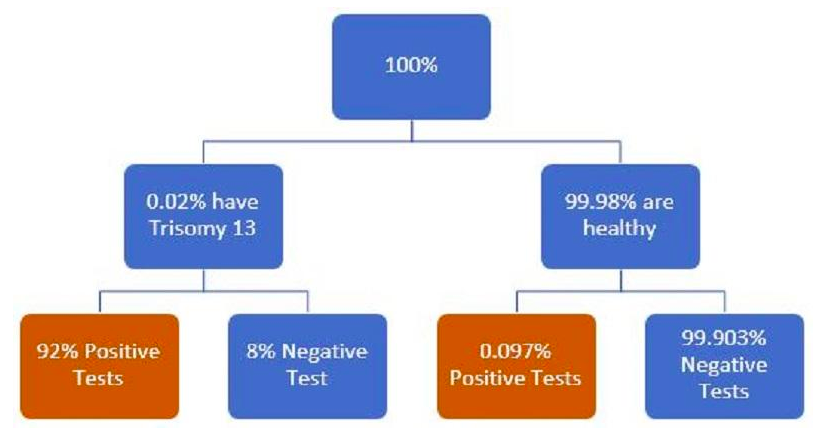 |
| (Please fill in the correct numbers) How many unborn children will get a positive NIPT test for Trisomy 13?  _____  Out of these, how many will *actually* have trisomy 13?  _____ | |

You just answered a question about how to understand a prenatal test for a genetic condition. How useful do you think this test is for expectant parents?

|  | Not at all useful | | | Moderately useful | | | | Very useful | | | | Extremely useful | | |
| --- | --- | --- | --- | --- | --- | --- | --- | --- | --- | --- | --- | --- | --- | --- |
|  | 0 | 10 | 20 | | 30 | 40 | 50 | | 60 | 70 | 80 | | 90 | 100 |

| This genetic test is: | 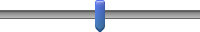 |
| --- | --- |

Please explain how you reached the answers you gave for the previous problem about genetic testing:

**Multiple Choice General Numeracy Scale**

From: Hill, W.T., Brase, G.L., Kenney, K. (2019). Developing a better and more user-friendly numeracy scale for patients. *HLRP: Health Literacy Research and Practice, 3*(3): e174-e180. https://doi.org/10.3928/24748307-20190624-01

Available at: <https://www.k-state.edu/psych/braselab/scales.html>

**Paper Folding Task**

From: Ekstrom, R.B., French, J.W., & Harman, H.H. (1976). *Manual for kit of factor-referenced cognitive tests*. Princeton, NJ: Educational Testing Service.

**Set Theory Test**

From: Brase, G.L. (2021). Which individual difference traits predict good Bayesian reasoning? Concurrent comparisons of underlying abilities. *Memory & Cognition, 49*(2), 235-248. https://doi.org/10.3758/s13421-020-01087-5

Available at: <https://www.k-state.edu/psych/braselab/scales.html>

**Graph Literacy Scale**

From: Galesic M., & Garcia-Retamero R. (2010). Graph literacy: a cross-cultural comparison. Medical Decision Making. 2011 May-Jun;31(3):444-57. https://doi.org/10.1177/0272989X10373805

**Verbal Cognitive Reflection Test (integrated)**

From:

Sirota, M., Dewberry, C., Juanchich, M., [Valuš](https://onlinelibrary.wiley.com/action/doSearch?ContribAuthorRaw=Valu%C5%A1%2C+Lenka), L., & Marshall, A. C., (2020). Measuring Cognitive Reflection without Maths: Developing and Validating the Verbal Cognitive Reflection Test. *Journal of Behavioral Decision Making, 34*(3), 3220343. https://doi.org/10.1002/bdm.2213

Thomson, K.S. & Oppenheimer, D.M. (2016). Investigating an alternate form of the cognitive reflection test, *Judgment and Decision Making, 11*, 99-113.

Items 1-10 come from Sirota, et al. (2020); Items 11-13 come from Thompson & Oppenheimer (2016). One item (marked with “**”) was common to both measures but included only once here.

1. Mary’s father has 5 daughters but no sons – Nana, Nene, Nini, Nono. What is the fifth daughter’s name probably? *correct answer: Mary, intuitive answer: Nunu*
2. If you were running a race, and you passed the person in 2nd place, what place would you be in now? *correct answer: 2nd, intuitive answer: 1st ***
3. It’s a stormy night and a plane takes off from JFK airport in New York. The storm worsens, and the plane crashes-half lands in the United States, the other half lands in Canada. In which country do you bury the survivors? *correct answer: we don’t bury survivors, intuitive answer: USA*
4. A monkey, a squirrel, and a bird are racing to the top of a coconut tree. Who will get the banana first, the monkey, the squirrel, or the bird? *correct answer: there is no banana on a coconut tree, intuitive answer: bird*
5. In a one-storey pink house, there was a pink person, a pink cat, a pink fish, a pink computer, a pink chair, a pink table, a pink telephone, a pink shower – everything was pink! What colour were the stairs probably? *correct answer: no stairs in a one-storey house, intuitive answer: pink*
6. How many of each animal did Moses put on the ark? *correct answer: none, intuitive answer: two*
7. The wind blows west. An electric train runs east. In which cardinal direction does the smoke from the locomotive blow? *correct answer: no smoke from an electric train, intuitive answer: west*
8. If you have only one match and you walk into a dark room where there is an oil lamp, a newspaper and wood – which thing would you light first? *correct answer: match, intuitive answer: oil lamp*
9. Would it be ethical for a man to marry the sister of his widow? *correct answer: not possible, intuitive answer: yes*
10. Which sentence is correct: a) “the yolk of the egg are white” or b) “the yolk of the egg is white”? *correct answer: the yolk is yellow, intuitive answer: b*
11. A farmer had 15 sheep and all but 8 died. How many are left? (intuitive answer: 7; correct answer: 8)
12. Emily’s father has three daughters. The first two are named April and May. What is the third daughter’s name? (intuitive answer: June; correct answer: Emily)
13. How many cubic feet of dirt are there in a hole that is 3’ deep x 3’ wide x 3’ long? (intuitive answer: 27; correct answer: none)

**Table S1**

*Experiment 1: Correlations between individual difference measures and Bayesian reasoning performances.*

|  | **Bayesian Reasoning** | **Numeracy** | **GraphLit** | **CRTverbal** |
| --- | --- | --- | --- | --- |
| Bayesian Reasoning | — |  |  |  |
| Numeracy | 0.251 *** | — |  |  |
| GraphLit | 0.128 ** | 0.363 *** | — |  |
| CRTverbal | 0.153 ** | 0.318 *** | 0.257 *** | — |
| Visuospatial | 0.233 *** | 0.485 *** | 0.304 *** | 0.340 *** |

* significant at the <.05 level; ** significant at the <.01 level; *** significant at the <.001 level

*Experiment 1: The results from binomial logistic regression analyses of Bayesian reasoning task performance, with different numerical formats.*

**Table S2**

Bayesian reasoning performance with information given as percentages.

| **Predictor** | **Estimate** | **SE** | **Z** | **p** |
| --- | --- | --- | --- | --- |
| Intercept | -12.1057 | 10.247 | -1.181 | 0.237 |
| Numeracy | 0.4244 | 0.927 | 0.458 | 0.647 |
| Visuospatial | 0.1914 | 0.376 | 0.510 | 0.610 |
| GraphLit | 0.0939 | 0.860 | 0.109 | 0.913 |
| CRTverbal | -0.1069 | 0.312 | -0.343 | 0.732 |

**Table S3**

Bayesian reasoning performance with information given as naturally sampled frequencies.

| **Predictor** | **Estimate** | **SE** | **Z** | **p** |
| --- | --- | --- | --- | --- |
| Intercept | -7.1472 | 1.3745 | -5.200 | **< .001** |
| Numeracy | 0.3609 | 0.1123 | 3.212 | **0.001** |
| Visuospatial | 0.0936 | 0.0424 | 2.206 | **0.027** |
| GraphLit | 0.0582 | 0.1129 | 0.515 | 0.606 |
| CRTverbal | 0.0369 | 0.0502 | 0.735 | 0.462 |

*Experiment 2: The results from binomial logistic regression analyses of Bayesian reasoning task performance, with different numerical formats.*

**Table S4**

Binomial logistic regression of Bayesian reasoning performance with information given as percentages.

| **Predictor** | **Estimate** | **SE** | **Z** | **p** |
| --- | --- | --- | --- | --- |
| Intercept | -199.342 | 53329.198 | -0.00374 | 0.997 |
| Numeracy | 17.609 | 4848.109 | 0.00363 | 0.997 |
| Visuospatial | 0.129 | 0.257 | 0.49945 | 0.617 |

**Table S5**

Binomial logistic regression of Bayesian reasoning performance with information given as natural frequencies.

| **Predictor** | **Estimate** | **SE** | **Z** | **p** |
| --- | --- | --- | --- | --- |
| Intercept | -5.148 | 0.8659 | -5.95 | **< .001** |
| Numeracy | 0.270 | 0.0958 | 2.82 | **0.005** |
| Visuospatial | 0.118 | 0.0310 | 3.79 | **< .001** |
